# Design and Evaluation Challenges of Conversational Agents in Health Care and Well-being: Selective Review Study

**DOI:** 10.2196/38525

**Published:** 2022-11-15

**Authors:** Ahmet Baki Kocaballi, Emre Sezgin, Leigh Clark, John M Carroll, Yungui Huang, Jina Huh-Yoo, Junhan Kim, Rafal Kocielnik, Yi-Chieh Lee, Lena Mamykina, Elliot G Mitchell, Robert J Moore, Prasanth Murali, Elizabeth D Mynatt, Sun Young Park, Alessandro Pasta, Deborah Richards, Lucas M Silva, Diva Smriti, Brendan Spillane, Zhan Zhang, Tamara Zubatiy

**Affiliations:** 1 School of Computer Science University of Technology Sydney Sydney, New South Wales Australia; 2 Centre for Health Informatics Macquarie University Sydney, New South Wales Australia; 3 Center for Biobehavioral Health The Abigail Wexner Research Institute Nationwide Children’s Hospital Columbus, OH United States; 4 IT Research & Innovation The Abigail Wexner Research Institute Nationwide Children's Hospital Columbus, OH United States; 5 Bold Insight UK London United Kingdom; 6 College of Information Sciences and Technology Pennsylvania State University University Park, PA United States; 7 College of Computing and Informatics Drexel University Philadelphia, PA United States; 8 School of Information University of Michigan Ann Arbor, MI United States; 9 California Institute of Technology Pasadena, CA United States; 10 Department of Computer Science National University of Singapore Singapore City Singapore; 11 Department of Biomedical Informatics Columbia University New York City, NY United States; 12 Steele Institute for Health Innovation Geisinger Danville, PA United States; 13 IBM Research - Almaden San Jose, CA United States; 14 Khoury College of Computer Sciences Northeastern University Boston, MA United States; 15 School of Art and Design University of Michigan Ann Arbor, MI United States; 16 Cognitive Systems Section, Department of Applied Mathematics and Computer Science Technical University of Denmark Kongens Lyngby Denmark; 17 Demant A/S Smørum Denmark; 18 School of Computing Macquarie University Sydney, New South Wales Australia; 19 Department of Informatics University of California, Irvine Irvine, CA United States; 20 ADAPT Centre University College Dublin Dublin Ireland; 21 School of Computer Science and Information Systems Pace University New York City, NY United States; 22 Georgia Institute of Technology Atlanta, GA United States

**Keywords:** conversational interfaces, conversational agents, dialog systems, health care, well-being

## Abstract

**Background:**

Health care and well-being are 2 main interconnected application areas of conversational agents (CAs). There is a significant increase in research, development, and commercial implementations in this area. In parallel to the increasing interest, new challenges in designing and evaluating CAs have emerged.

**Objective:**

This study aims to identify key design, development, and evaluation challenges of CAs in health care and well-being research. The focus is on the very recent projects with their emerging challenges.

**Methods:**

A review study was conducted with 17 invited studies, most of which were presented at the ACM (Association for Computing Machinery) CHI 2020 conference workshop on CAs for health and well-being. Eligibility criteria required the studies to involve a CA applied to a health or well-being project (ongoing or recently finished). The participating studies were asked to report on their projects’ design and evaluation challenges. We used thematic analysis to review the studies.

**Results:**

The findings include a range of topics from primary care to caring for older adults to health coaching. We identified 4 major themes: (1) Domain Information and Integration, (2) User-System Interaction and Partnership, (3) Evaluation, and (4) Conversational Competence.

**Conclusions:**

CAs proved their worth during the pandemic as health screening tools, and are expected to stay to further support various health care domains, especially personal health care. Growth in investment in CAs also shows the value as a personal assistant. Our study shows that while some challenges are shared with other CA application areas, safety and privacy remain the major challenges in the health care and well-being domains. An increased level of collaboration across different institutions and entities may be a promising direction to address some of the major challenges that otherwise would be too complex to be addressed by the projects with their limited scope and budget.

## Introduction

Conversational agents (CAs) are applications that facilitate human-computer interaction through natural language. Automatic speech recognition (ASR) and natural language processing (NLP) models help to interpret human language and produce appropriate responses [1]. CAs (also widely known as chatbots, virtual assistants, dialog systems, or voice assistants) are used in several domains such as e-commerce, scheduling services, and question-answer systems [2]. The user-system interaction could be over text (eg, SMS text messaging over an app or web service), voice (eg, interactive voice response via phone calls, voice assistants via a smartphone or smart speaker), or multimodal (eg, visual, text, and audio feedback and interaction via a smartphone, smart speaker, or any other smart and internet of things devices) [3].

CAs have been variously used and studied in health care for supporting behavioral health and healthy living [3,4]; health information seeking [5-9]; appointment, medication, symptom tracking and chronic condition management [10-12]; and facilitating COVID-19 screening and information sharing [13,14]. Mobile phone ownership enables and increases the potential applications, availability, and access to CAs in practice. Mobile phone ownership is around 15 billion worldwide as of 2021 [15], and 97% of US adults own a mobile device [16]. Current studies and randomized trials showed that CAs could be effectively used in health care delivery and improving health outcomes, such as improving mental health [17], maternal health [18], and healthy behaviors [19]. In addition, there is an increasing investment in chatbots in the health care industry; some examples include Woebot, Babylon, and ADA Health [20].

Despite the increasing interest in using CAs in supporting health care and well-being, there are many challenges in the development, deployment, and use of CAs. Recent review studies have highlighted some of the challenges including NLP [21,22], patient safety [1,23,24], integration with other technologies [22], information dissemination [25,26], medico-legal issues [27], and ethics [28]. In response to that, recent workshops have explored the challenges and opportunities of conversational user interfaces [29-31] and the design and evaluation of CAs in health care [32]. Here we contribute to this developing literature by reporting a self-assessment of 17 such projects.

In May 2020, a workshop entitled “Conversational Agents for Health and Well-being” was held at the *ACM (Association for Computing Machinery) Conference on Human Factors in Computing Systems (CHI 2020)* [32]. The aim was to understand the most current challenges recent research projects face and devise potential directions for future research to address those challenges. The workshop included completed or ongoing projects from 30 participants in 5 countries, covering various topics from supporting older adults to mental health and coaching to supporting everyday health. Following the workshop, participants were invited to collectively report the design and evaluation challenges of CAs in health care to provide researchers, designers, and health care professionals practical perspectives on these challenges. This paper aims to present the challenges of designing and evaluating CAs derived from recent health care projects conducted in the last 2 years.

## Methods

We followed a selective review study design focusing on the challenges of recent studies on CAs in health and well-being. Coauthors were invited to report their original CA research in health care and well-being, outlining major challenges in the design and evaluation of the CA being used in their research. Coauthors were asked to report on design and evaluation challenges they faced in their project. In their written report, each coauthor or author group (1) described their research, (2) explained challenges (limited to 3 major design challenges and 3 major evaluation challenges), (3) explained how they addressed the challenges or how they plan to address, and (4) support their findings and suggestions with prior literature. Only the information provided through the written report was analyzed as a case study. Each case study went through an open peer review process among authors and was revised. Finalized cases were analyzed by 3 coauthors (ABK, ES, and LC). We used thematic analysis to identify, assess, and analyze the patterns in the cases [33]. The following steps were used in the analysis process: (1) familiarizing with the data, (2) generating initial codes, (3) searching for themes, (4) reviewing and refining themes, (5) defining and naming themes, and (6) reporting the findings (Textbox 1).

**Steps used in the analysis process.**

1. Familiarizing with data
  - To gain familiarity with the data and understand the depth and breadth of the content, coauthors (ABK, ES, and LC) read and re-read the case studies.
2. Generating initial code
  - Following an open-coding approach (without predefined codes, developed, and modified during the coding process), coauthors (ABK, ES, and LC) created initial codes independently. They reviewed the codes iteratively. The codes were compared, and group decisions and consensus created the finalized codes. Coauthors used Google Sheets to create codebooks.
3. Searching for themes
  - The codes were sorted at first to understand the frequency of occurrence. They were reviewed to find patterns and grouped into the themes collectively by the coauthors (ABK, ES, and LC). Each theme was labeled to guide the grouping and reviewed by the coauthors (ABK, ES, and LC) iteratively. Similar to the coding, coauthors reached a consensus to finalize themes.
4. Reviewing and refining themes
  - All themes were reviewed. The relationships between codes and themes were discussed by the coauthors (ABK, ES, and LC). Some of the themes were combined that were found to be related; for example, obtaining domain information and training data were combined into Domain Information and Training. Themes were reviewed and finalized for consistency regarding their content, by consensus.
5. Defining and naming themes
  - Definitions of the themes were created regarding codes, subthemes, and corresponding cases. In case of disagreements, coauthors reviewed themes to ensure consensus in theme content, definition, and labeling.
6. Reporting the findings
  - Thematic analysis results were reported through a chart with themes, subthemes, definitions, associated cases, and frequency of occurrences.

## Results

### Overview

The review included 17 studies covering many domains from primary care to caring for older adults to health coaching. A summary of the projects is presented in Table 1, with a unique project number for each project to be referenced in the presentation of results. The thematic analysis identified 4 major themes: (1) Domain Information and Integration, (2) User-System Interaction and Partnership, (3) Evaluation, and (4) Conversational Competence (Table 2).

**Table 1:** A summary of participating projects, including titles, domain, CA^a^ purpose, and CA input and output modalities.

Project number	Project title	Health/well-being domain	CA purpose	CA input/output	Project status
1	Digital Scribe: A Wizard of Oz Study	Primary care	Work with general practitioners to document patient information in a consultation	Written and spoken/written, spoken, and visual	Ongoing
2	Speech Diversity and Speech Interfaces – Considering an Inclusive Future Through Stammering [34]	Accessibility and inclusivity/speech diversity	All speech-based CAs (nonspecific projects)	Spoken/written, spoken, and visual	Ongoing
3	ADELE: An Artificial Conversational Care Agent for the Elderly [35-37]	Care of the elderly in their homes	Provide health and well-being care, advice, and monitoring	Written/written	Completed
4	Talk the Talk: How Human Conversational Agents Build Trust [38-41]	People with visual impairments	Support navigation and other activities by having the person with visual impairment share their smartphone camera feed. Based on the camera feed and conversational interaction, plus use of online tools such as Google Maps, a remotely located sighted person provides guidance to the person with visual impairment.	Spoken/spoken	Ongoing
5	Empowering Older Adults With Mild Cognitive Impairment and Their Caregivers Using Conversational Agents	Older adults with MCI^b^	Empower the person with MCI as well as their caregiver; amplify caregiver	Spoken/spoken and visual	Ongoing
6	Adaptive Conversational Agents for the Health Care Enterprise	Health care enterprise, payer	Help users answer analytical questions about health care enterprises	Written and spoken/written, spoken, and visual	Completed
7	Encouraging Users’ Self-disclosure With a Chatbot Mediator [42-44]	Mental health care	Collect truthful self-disclosure and deliver guidance	Written/written	Ongoing
8	Motivational Interviewing Conversational Agent (MICA) [45,46]	Family eating habits	Deliver a counseling method named motivational interviewing in an automated manner to help parents eat healthier along with their children	Spoken/spoken	Completed
9	Conversational Agent for African Americans With Chronic Illnesses [47,48]	Chronic illness	Deliver health information on COVID-19 to African Americans with chronic illnesses	Written/written	Completed
10	Exploring Voice Assistants in Multimodal Food Journaling [49,50]	Food journaling	Entries in food journal	Spoken/written and spoken	Completed
11	t2.coach: A Chatbot Health Coach for Diabetes Self-management [51,52]	Health coaching	Health coaching and goal setting for type 2 diabetes self-management	Written/written and visual	Ongoing
12	Designing Audiologist Bots Fusing Soundscapes and User Feedback	Hearing health care	Recommending personalized hearing aid settings by gathering user feedback in real-world environments	Written and spoken/written, spoken, and visual	Ongoing
13	Utilization of Self-Diagnosis Health Chatbots in the Wild: A Case Study [53]	Self-diagnosis	Offering medical advice (eg, diagnostic suggestions) to patients based on their input (eg, symptoms)	Written and spoken/written	Completed
14	eADVICE: Providing Specialist Treatment Advice to Patients on Waiting List [54]	Children and their families referred to incontinence and sleep clinic awaiting appointment	Discuss treatments to encourage adherence	Written/written and spoken	Ongoing
15	Symptom and Health Events Tracking at Home for Children With Special Health Care Needs (CSHCN) Using Conversational Agents [55,56]	Documentation and care coordination support for children with special health care needs	Assisting caregivers and patients with tracking and communicating symptoms and health events outside of clinical settings to reduce documentation burden and facilitate care coordination	Written and spoken/written and spoken	Completed
16	Palliative Care With Spiritual Support by Conversational Agents	Elderly care, spiritual support, human-agent/robot interaction	Development of a CA that provides end of life planning and spiritual counseling	Written/spoken and visual	Ongoing
17	HarborBot: A Chatbot for Social Needs Screening [57,58]	Social needs screening in public hospital emergency departments	Collect high-quality social needs data from vulnerable populations while increasing engagement	Written/written and spoken	Completed

^a^CA: conversational agent.

^b^MCI: mild cognitive impairment.

**Table 2:** A summary of themes and subthemes and the studies reporting them.

Themes and subthemes		Reported by	
**Domain Information and Integration**			
	Domain Information and Training	P2^a^, P6, P10, P11, P13, P14, P17	
	Integration and Infrastructure	P11, P14, P15	
**User-System Interaction and Partnership**			
	Personalization	P3, P5, P6, P8, P9, P11, P12, P13, P14	
	Relationship Building	P3, P4, P7, P12, P13, P16, P17	
	Safety and Privacy	P3, P5, P7, P8, P9, P10, P13	
	User Engagement	P4, P7, P9, P10, P13, P14	
**Evaluation**			
	Methodological Limitations	P1, P8, P11, P12	
	Experimental Limitations	P1, P3, P5, P12, P13, P14, P17	
	Lack of Guidance on Evaluation	P1, P2, P9, 13, P15	
**Conversational Competence**			
	Topic Detection and ASR^b^	P1, P2, P3, P8, P10, P12, P15	
	Discoverability and Conversational Interaction Model	P10, P11, P1, P2, P17	
	Accessibility and Inclusivity	P2, P5, P8, P9, P14, P16, P17	

^a^P: project.

^b^ASR: automatic speech recognition.

### Domain Information and Integration

#### Overview and Subthemes

Health care CAs often operate within particular domains of health care that require the integration of domain-specific information and language. This theme is concerned with the challenges of obtaining the required problem domain information, collecting data to train CAs, and integrating CAs with the existing systems and infrastructures. For example, a CA to be designed for helping general practitioners in the primary care domain needs to be trained by a large number of doctor-patient conversations. Obtaining this kind of information is challenging and resource intensive. In addition, the expert medical knowledge needs to be translated into conversational format. Similarly, CAs cannot be developed as isolated applications: they need to be integrated with the existing systems and infrastructure.

#### Domain Information and Training

Two projects (P6 and P14) reported on the difficulties of obtaining domain information, in these cases, for health care enterprise and medical adherence communications. These difficulties are associated with the lack of time of domain experts (P6), the domain information being not in a conversational format (P6) or being distributed across many subdomains (P6), and the knowledge acquisition bottleneck (P14). While P6 recruited subject matter experts to review conversation flows and response frames to format health care data for conversation-like interactions, P14 implemented incremental acquisition from the expert or user to alleviate the problem of obtaining domain-specific information.

#### Integration and Infrastructure

Three studies reported some challenges associated with integrating CAs into existing infrastructures. One project (P15) using CAs to support health care tracking of children with special needs at home explained the security and privacy challenges of integrating CA-collected information into clinical systems and workflows. Using interoperability standards (eg, Fast Healthcare Interoperability Resources) to share caregiver notes with health care professionals was suggested as one of the fundamental approaches for integration (P15). Many CAs are developed using underlying development platforms such as Amazon Alexa Skills or Google Dialogflow. P14 highlighted the difficulty in adapting to the rapidly evolving platforms that can potentially make the CAs designed according to the previous platform functionality stop working. Finally, P11 discussed the tension between the ubiquity and richness of the underlying platforms. For example, while SMS-based text messaging is ubiquitous, it is purely text based and does not offer some of the multimodal interaction options of other messaging platforms such as Facebook Messenger or WhatsApp (eg, 1-click suggested response buttons or carousel menus). P11 used SMS text message–based messaging to support a higher degree of accessibility and ubiquity; however, to expand the richness of the user experience they included more immersive education content with multimedia messages including infographics to elaborate on each health goal option.

### User-System Interaction and Partnership

#### Overview and Subthemes

This was one of the major themes that captured several challenges related to the characteristics and qualities of interaction between users and CAs, and the ways in which users and CAs work together. The subthemes included Personalization of CAs, Relationship Building Between CAs and Users, Safety and Privacy, and User Engagement. Some common challenges were supporting users’ trust in CAs, enabling CAs to show empathy, and ensuring users’ privacy.

#### Personalization

Personalization emerged as an essential design feature as part of the projects. Personalization was reported as a challenge in 10 projects. The personalization challenges were providing appropriate responses based on users’ context (P8 and P3), tailoring conversations for different user groups with a broad age range and different health literacy levels (P14, P6, P9, P11, and P12), and minimizing the question overload in surveys (P13). While 1 project reported on the difficulties in evaluating the effects on adaptive features (P6), another project presented the challenges associated with designing for dyads (P5), which include a patient and a caregiver. P5 explained that dyads using the same device pose several challenges, including dyads’ difficulties managing technology, dyads’ overall wide range of technology literacy, and how to support both members of the dyad through the functionality of the CA.

#### Relationship Building

Humans and CAs may have different forms of relationships ranging from very short-term relationships typically characterized by one-off task-based conversational exchanges to long-term relationships in which CAs have longer conversational interactions across different topics over a longer period. Relationship building—how a human-CA relationship is established and maintained—was reported as a challenge in 13 projects. Trust (P3, P4, P7, P13, and P16), empathy (P4, P17, and P16), self-disclosure (P7), and transparency (P12) were presented as important dimensions of relationship building. One project focusing on designing a CA for people with visual impairment (P4) explains the importance of CAs to show empathy in establishing and strengthening trust between the sighted Aira agent and the client with visual impairment. According to P4, to incorporate empathy into conversational interaction, an agent must represent not only the situation itself (eg, what objects are present, their spatial relationships, movement vectors) but also the other agent’s experience and interpretation of the situation. A project designing a CA for older adults’ care (P3) describes the difficulties of building trust due to concerns about security, legal issues, the sharing and storing of personal and sensitive information, and privacy and ethical concerns, among others [59]. Issues with any of these and other concerns have the potential to damage the relationship critically or terminally between the CA and the patient. To address this challenge, P3 incorporated trust-building and repair strategies from the onset. Previous research has shown that reliability is likely the preeminent factor in building trust between the patient and the artificial care agent [60,61]. By contrast, other factors such as competence, benevolence, and integrity are also likely to significantly influence [62]. Transferring users’ self-disclosure was reported as a challenge in human-in-the-loop artificial intelligence systems where CAs mediate between a user and a domain expert (P7).

#### Safety and Privacy

Safety of user data and privacy of personal information shared with CAs have influenced the user decision and perception toward CAs. Six projects reported users’ safety (P3 and P13) and privacy (P5, P7, P8, and P10) as challenges. P10 explained that while some users were worried that using the voice assistant would disturb others, other users felt discomfort or concerned with privacy when other people could hear them track their food. To tackle this issue, some suggested solutions in P10 included adopting other devices or modalities for input (eg, taking picture or text input using phone or web), as well as implementing food “template” features for quick commands (eg, saying “Alexa, journal number 1” or “Google, journal same breakfast as yesterday”). In the cases of CAs as mediators between 2 users (P7) or multiple users (P5), privacy challenges may become more critical. As many health care CAs deal with safety-critical user information and decision making, P3 and P13 noted the need for more strict and standardized evaluation measures for CAs.

#### User Engagement

Establishing user engagement strategies for CAs is fundamental to improving user experience and sustained use. However, 6 projects reported enabling and maintaining user engagement as a challenge (P4, P7, P9, P10, P13, and P14). P9 explained the importance of culturally sensitive CAs to support increased trust and adoption in the context of helping African Americans with chronic diseases and the difficulties of understanding and incorporating cultural aspects into CAs. P10’s focus on creating a multimodal food journal mentioned their participants’ problem of remembering or discovering the voice commands to track their food, and the need to have a better mapping of commands to multiple utterance styles and advanced intent recognition. P13, a project using a self-diagnosis chatbot, reported that their users tended to drop out of the consultation with their chatbot, especially during the early stages, and pointed out the importance of examining and evaluating the mechanisms and approaches that can increase the uptake and utilization of health chatbots. P14 incorporated various strategies into the chatbot to support higher treatment adherence for pediatric patients awaiting a specialist appointment. These included developing a working alliance; having face-to-face communication; using everyday conversational language; and empathic language strategies such as choices, consequences, and nonjudgmental affirmations.

### Evaluation

#### Overview and Subthemes

The Evaluation theme encapsulates 3 limitations that authors have encountered during their experiments with CAs: (1) methodological limitations that show challenges in evaluating interactions and performance of CAs; (2) experimental limitations related to the challenges with data collection and analysis and study environment; and (3) lack of guidance in evaluation describing challenges to navigate assessment of CAs without guidance or prior evidence.

#### Methodological Limitations

By nature, CA interactions are designed to communicate with humans and are dependent on intellectually crafted bidirectional conversations that are hard to generate or replicate. Early efforts toward testing the CAs included scripted conversations to measure the performance of CAs objectively. Yet, scripted conversations fall short in mimicking the actual number of conversations and iterations that may occur in the real world (P1). In addition, a limited number of interactions create a barrier to evaluating the performance effectively. Creating complex scripts or testing with actual patients are some of the solutions proposed (P1).

To provide standard measures to evaluate the performance of CAs, user-CA interactions can be tested in a controlled environment using scripts, such as scenarios, role-playing, or Wizard of Oz testing. However, these scripted or simulated interactions might be limited to providing organically flowing conversations (P1) and accurate assessments due to the simulated nature of system functionalities and user scenarios (P8). Such evaluation yields the results only in a controlled environment, impacting the end user’s judgment (P8). The observations on user-CA interactions may explain a CA’s efficacy to a degree yet not its effectiveness, which can be observed in the real-world environment. Using actual end users with different health and well-being needs in real-world settings and observing them longitudinally may improve the evidence of CA interactions (P1, P11, and P14).

#### Experimental Limitations

##### Effects of Training Materials/Unplanned Events

Training data are core requirements in developing and assessing CAs performance. However, it could be hard to gauge the effects of training materials (P5). Developers must track the features used in training and compare them with the actual CA usage. Similarly, unplanned or unforeseen events can affect the outcomes of CA interaction evaluations. The COVID-19 pandemic shifted the norms toward remote management of experiments, causing miscommunications and inefficiencies in simple user training and troubleshooting (P5).

##### Accessing Vulnerable Populations

It is important to design for diverse populations, including vulnerable populations, and people with various social ills, such as homelessness, poverty, and hunger [58]. However, recruiting and engaging them with limited resources and connections with community partners can be difficult. In terms of improving CA interaction with such populations and enhancing access to the CA, inputs from heterogeneous groups and different users are needed through alternative platforms and technologies (P12 and P14). For instance, CA use evaluation in an emergency department with low-literacy users may require trained personnel to guide users and understand their experiences (P17).

##### Challenges of Testing in Real-World Settings

In research, test settings and iterations often occur in controlled environments, without real-world interactions to objectively assess CAs. This results in decontextualization of testing and yields limited results. Some significant difficulties are involved in performing CA testing in real-world, authentic settings. For instance, P1, in a study with CA-supported automated documentation, reported that without a real-world electronic health record interaction, many work routines cannot be tested. Even if a CA gets integrated into electronic health record, technically, logistically, and legally it is hard to roll out. However, such efforts are necessary for understanding all end users’ perceptions (nurses, doctors, patients) and the complexity of medical workflow (P17). P3 suggested a staged evaluation approach through a research platform, which will allow evaluations in mock care settings. There are still very few user-CA evaluations in real-world settings. Further efforts are required to promote real-world testing (in uncontrolled authentic environments). P13 explained that without an in-depth understanding of contextual elements in a problem domain, it is challenging for CA designers and developers to figure out how to improve the user experience and how to overcome the challenges in the actual use of health care CAs.

#### Lack of Guidance in Evaluation

##### Lack of Evaluation Data for Special Population Groups

As with vulnerable populations, there is a lack of evaluation data for special population groups. This issue may lead to having no real knowledge of user experiences with CAs for people with diverse speech patterns (P2). Similarly, there is a lack of evaluation material designed (P13) specifically for marginalized or minor user groups, such as African Americans with low technology literacy (P9). Development of population-specific evaluation methods and promotion of participatory design and interactive sessions are necessary (P2 and P9).

##### Lack of Evaluation Guidelines and Metrics

The lack of evaluation guidelines for CAs leads to creating robust frameworks for effectively and uniformly measuring impact and outcomes. In addition, the CA-based interventions’ safety, efficacy, and effectiveness are lacking due to no clear guidance provided in the literature yet (P13). Metrics to measure the effects of interaction, engagement, and measures for health outcomes are necessary. One solution could be longitudinally observing interaction to identify key indicators to be used as success metrics (P15).

Theoretical approaches to evaluating the CAs may not be aligned with the standard measures. In P11, behavior change techniques showed a mismatch with usability metrics, potentially showing an inverse relationship between user engagement (quality of communication in user experience and CA usage patterns) and behavior change techniques (eg, goal setting). Combining theories and evaluation approaches might be necessary to create multifaceted evaluation metrics and triangulate use patterns.

##### Difficulties in Multimodal Testing

CA could be potentially provided in multiple platforms and different modalities (eg, text-based chatbot, voice assistant with avatar). Evaluating individual modalities in a multimodal system is necessary, yet it is hard to evaluate the graphical and conversational interfaces separately. However, it might still be possible to design visual elements and layouts in a minimal way to reduce their effects on users’ perception and assessment of the CA interface (P1).

### Conversational Competence

#### Overview and Subthemes

Several projects discussed challenges related to the conversational competence of CAs and the impacts these might have on people’s interactions. Competence here refers to accurately understanding user input and responding appropriately, whether the conversation is an appropriate metaphor for design and how user interactions can be best facilitated, and how CA interactions can be made more accessible and inclusive.

#### Topic Detection and Automated Speech Recognition

Detecting the topic being discussed can be difficult in several CA scenarios. The unscripted nature of the social talk, often undertaken as part of caregiving interactions, makes it difficult to follow the discussed topics (P3). Even more scripted interactions such as primary care consultations may be nonlinear and fragmented, creating further difficulties in detecting the current topic being discussed (P1). Proposed solutions include advanced topic detection methods (P3) and collaboratively built data sets to support these methods (P1). Using specific phrases to highlight topic shifts during interactions (P1) and personalized systems (P3) may also improve CAs.

Topic detection in CA in health care settings also faces challenges related to specific contexts and users. For example, tracking health care outcomes for different patients and treatments requires an understanding of specific medical terms (P15). Using services such as Amazon Comprehend Medical [63] alongside manual interventions could help expand appropriate vocabulary to improve CA comprehension. Similarly, P12 describes using CAs for people with hearing aids that may require them to use an agreed set of terms or additional supervised training to recognize “audiological intents.”

More generally, there is an ongoing challenge of understanding when someone has finished an utterance while communicating with a CA (endpoint detection). This may require additional research for people with diverse speech patterns such as stammering (P2), to collect the necessary audio data and understand their interactions.

In addition to detecting topics in interaction, CAs in health care interactions face difficulties with ASR. This is also common in CA interactions outside of health care [64]. Audio may be impacted by recording quality ambient noise (eg, other devices such as televisions or other people talking), which could be combated with the use of directional microphones (P1 and P15) [21]. Again, in more nuanced interactions such as using CAs for people with hearing aids, optimal CA responses may require the processing of environmental information such as loudness and signal-to-noise ratio (P12). Inserting such information into the dialog may improve CA performance for this type of interaction.

In addition to audio quality, the language a user produces can also be a limiting factor for CAs. Active error correction by a CA may help in the misunderstanding and nonrecognition of specific terms (P10). For privacy-first interactions, processing of speech data may be performed locally on the device rather than requiring any interaction with servers, though this may reduce the performance of the CA’s speech processing capabilities (P8).

#### Discoverability and Conversational Interaction Model

A key challenge in CA design is making the set of possible actions or commands discoverable for users [65]. Understanding and remembering how to interact with CAs in different contexts can create difficulties for people using these systems (P10 and P11). Making CAs open ended and lightweight may allow people to explore systems’ capabilities and adapt them for their purposes (P10). For people with low levels of technology literacy, CA-initiated dialogs can be implemented at consistent intervals to counteract the lack of discoverability of user-initiated features (P11).

CAs rely on turn-taking–based conversational communication and interaction models; however, they may not always be appropriate or require additional scaffolding. General practitioners taking notes using a CA, for example, may need to interrupt interactions with their patients. By contrast, screen-based technologies for the same task can support multimodal, more continuous interactions, and less intrusive data entry (P1). Continuing the multimodal use of information entry for general practitioners may improve or resolve interruptions. Furthermore, monitoring utterances in user-CA interactions may not be as smooth as human-human interactions. Consequently, enabling a CA to detect when someone has finished speaking is critical to more seamless interactions (P2). For diverse speech patterns, such as stammering, we may be able to draw on advice for interacting with various demographics, though crucially, we must understand the nuances of these interactions and design CAs with inclusivity in mind. For scenarios such as filling in forms and surveys with CAs, audio may be an inefficient modality and create design tension with sequential questions and wait times (P17). Optimizing delay usage to limit unnecessary wait times, using shorter phrases, and allowing people to opt-out of audio in multimodal CAs can be used to work against these limitations.

#### Accessibility and Inclusivity

Several projects discuss the need to make CAs accessible to a broad set of demographics or to focus on improving systems to make them inclusive for specific types of people. P14 identifies the need to provide equitable and easy access to CAs that improve health and well-being. They propose allowing access through a web browser and downloadable software, as well as offering technical support and downloadable fact sheets to work with or replace their CA. Off-the-shelf CAs may present difficulties for people in lower-resource areas (eg, limited internet access, financial constraints), which could be improved with systems that use more offline resources (P8), though this may present its own performance challenges that need to be addressed.

Levels of education, experience, and technological literacy can also impact users’ interactions with CAs (P5, P16, and P17). These obstacles may be overcome by providing multiple modalities to compensate for people’s preferences and abilities (P5 and P17) and engaging in participatory design with target demographics (P16). Evaluating such systems may require redeveloping protocols to include people with lower literacy and ensure they are able to understand the questions being posed to them (P17). Specific communities may also require additional thought in designing and evaluating CAs and building on the available sparse research literature (P2 and P9). Working with people who stammer, for example, has seen little research with CA interactions and requires a fundamental understanding of the barriers to successful interactions and how these can be overcome (P2). P9 highlights the need to consider African American communities and how their perceptions towards CA and interactions with them cannot be assumed to be the same as other communities. Consequently, novel methods and evaluation techniques may need to be developed with diverse populations in mind.

## Discussion

### Principal Findings

This review highlights numerous challenges of CA interactions in the health care and well-being fields, including 4 major themes on Domain Information and Integration, Conversational Competence, User-System Interaction and Partnership, and Evaluation. Many challenges reported in this review echo those discussed in related CA work. ASR errors are long-standing concerns with speech-based CAs [66] that can lead users to alter their speech patterns to increase comprehension [67]. Difficulties in fostering user engagement have been highlighted when CAs cease to perform their intended utility [68]. Existing research has addressed this issue, for example, by explaining the cause of interaction errors to users or allowing users to halt interactions when they desire [69]. Accessibility concerns discussed in this review also map to those discussed in prior work on both text-based [70] and speech-based CAs [71]. Research continues to identify how CA accessibility can be improved [72] and design recommendations such as the Web Content Accessibility Guidelines [73] have been suggested as a means of addressing these. Prior studies also reported challenges associated with involving and engaging patients in their home environments [74], limitations in short-duration laboratory studies and challenges in longitudinal assessments [75], and difficulties with multicomponent system evaluation [76].

In addition to the similar challenges identified in prior CA literature, this review identifies some challenges that are specific or more critical to health care and well-being domains, including empathy, safety, recruitment of vulnerable populations, and challenges in testing authentic settings. Prior work has brought attention to open challenges across the broader CA field [77] and highlights the need to create responsible CAs that focus on fairness, transparency, and ethics. This is critical for CAs discussed in this review, particularly given the sensitive nature of interactions and the underlying data collected from these. The fragmented nature of speech-based CA work, in particular, has recently been addressed [29,78]. This prior work notes a scarcity of robust evaluation metrics and research involving real-world testing. Our review shows that there are similar problems for application of CAs in health care and well-being. However, the nature of these domains means there can be additional obstacles when entering research phases that require strict considerations of medical ethics, laws, and standardized practices. Improving consistency in CA research and implementation may benefit from reaching out to other disciplines (eg, cognitive sciences, linguistics) [78], combining existing theories and evaluation approaches (see the “Lack of Guidance in Evaluation” section), and examining guidelines from both academia and industry [79]. While broad challenges around CAs must be considered within health care and well-being contexts, this review also identifies more nuanced challenges for these domains. Future work should consider how these different challenges overlap, depending on both the expected CA scenarios and user demographics.

### Data Collection in Health Care Domains

Obtaining domain-specific information and collecting data are typically challenging. There are 2 major reasons: first, health care professionals who can provide critical domain information are very time-poor and their contribution to the projects is limited; and second, health care data are extremely sensitive, and there are a lot of privacy and safety concerns. As recruiting a full-time health care professional is not financially feasible, an incremental acquisition method might be useful (P14). Data privacy and safety are significant concerns for health care professionals, and consumers are more hesitant to share information compared with other application areas (P1). For example, the Digital Scribe project needed to collect thousands of doctor-patient conversations in primary care settings to train their NLP algorithm [21]. However, managing the audio recording system, informing patients and getting their consent, and privacy and safety concerns of not only patients but also doctors made the data collection process extremely challenging. In this case, multi-institution collaborations are needed to organize and manage data collection to reduce the frictions in the process such as employing a dedicated technical person to provide support, more automated and easy-to-control audio recording system, and streamlined patient consent gathering. Such data collection challenges are not specific to primary care as many health care settings have similar situations and requirements. Therefore, incentivizing collaborations and data sharing between institutions and creating ethical frameworks to facilitate data sharing are needed, with some already emerging in the CA community [80]. In addition, proven data safety protocols and certifications can be made available to ensure safety and increase user trust. Integrating CAs into existing systems and enabling data sharing across systems were other challenges reported. Fast Healthcare Interoperability Resources was suggested as a promising interoperability standard (P15).

### Developing Empathy in CA Interactions

Concerning the theme User-System Interaction and Partnership, empathy, safety, and privacy emerged as important challenges. Especially for CAs used in mental health applications, being able to show empathy is a valuable characteristic. Challenges related to empathy can be grouped into 2 categories: challenges of detecting the users’ current emotional sensitivity to a topic and crafting an appropriate response to the user according to their situation and preferences. For example, P4, using their CA interface Aira, used client profiles (typical interaction contexts, preferred measurement units) to reference and incorporate these details in the interaction to signal empathy. P17 aimed their CAs to show empathy through more minimal means. This contrasts with other successful but more costly implementations of embodied CAs [81,82] in general medical contexts. P17 designed a series of neutral and empathetic reactions to user answers as well as other social utterances to augment the question administration dialog. They included phrases that help the user anticipate a sensitive topic before it is introduced (eg, “The next questions are about your personal safety and may be tough to answer.”), provide acknowledgments for answering neutral questions (eg, “Okay, I’m getting a better idea of where you are at,” “Got it”), and empathetic reactions to sensitive questions (eg, “That must be stressful, I’m sorry to hear that.”). They also pointed out some users’ different preferences, such as a lower level of socialization in chat than others.

### Establishing Safety and Privacy in CA Applications

Safety and privacy concerns are more critical in health and well-being applications. A systematic review found that patient safety concerns were rarely addressed in the majority of the papers reviewed [1]. Similarly, Bickmore et al [24] found that CAs with unconstrained natural language input pose serious safety risks. Prior studies also found that commonly available voice assistants fail to answer appropriately to safety-critical user prompts [4,6]. Safety risks may occur due to misrecognized prompts, the inability to detect the severity of users’ prompts (when there are no ASR errors), or gaps in clinical reasoning. A 5-stage framework for evaluating different aspects of symptom checkers might be adapted to be used for other CA applications [83]. Privacy of personal user data is also of utmost importance. Personal health data are considered one of the most sensitive types of information. Therefore, protecting the privacy of users’ data becomes even more critical in health care CAs. Data privacy is particularly more relevant to CAs as many CAs process user prompts through third-party services in the cloud. Thus, there is a valid concern about the security of such information. Local NLP engines might be a solution to this (P8).

### Limitations

This review included a limited number of studies in the emerging domain of health care and well-being CAs, which were conducted within a specific period and reported during a conference workshop. The main purpose is to provide a snapshot of some of the major challenges faced by the recent projects in this area rather than providing a comprehensive overview of the challenges. Although the reported challenges provide a useful overview of some significant challenges, they should not be considered the complete set of challenges in this domain. They represent the challenges faced by some recent projects conducted by the researchers actively working in this area across the globe.

### Conclusion

This paper examined 17 recent studies of CAs in health care and well-being to identify design and evaluation challenges. While many challenges, including accessibility, personalization, and empathy provision, are shared with other application areas of CAs, safety and privacy remain the major challenges that are more critical in the health care domain.

CAs proved their worth during the pandemic as health screening tools, and are here to stay to further assist personal health care. Growth in investment in CAs also shows the value as a personal assistant. An increased level of collaboration across different institutions and entities may be a promising direction to address some of the major challenges that otherwise would be too complex to be addressed by the projects with their limited scope and budget.

## Abbreviations

ASR: automatic speech recognition
CA: conversational agent
MCI: mild cognitive impairment
NLP: natural language processing
